# SARS-CoV-2 Post-Infection and Sepsis by *Saccharomyces cerevisiae*: A Fatal Case Report—Focus on Fungal Susceptibility and Potential Virulence Attributes

**DOI:** 10.3390/tropicalmed8020099

**Published:** 2023-02-02

**Authors:** Lívia S. Ramos, Luca Mokus, Heloisa F. Frota, Marcos V. Santos, Simone S. C. Oliveira, Manoel M. E. Oliveira, Gisela L. Costa, Ana Luísa Alves, Andréa R. Bernardes-Engemann, Rosane Orofino-Costa, Ana Carolina Aor, Marta H. Branquinha, André L. S. Santos

**Affiliations:** 1 Laboratório de Estudos Avançados de Microrganismos Emergentes e Resistentes (LEAMER), Departamento de Microbiologia Geral, Instituto de Microbiologia Paulo de Góes (IMPG), Universidade Federal do Rio de Janeiro (UFRJ), Rio de Janeiro 21941-902, Brazil; 2 Programa de Pós-Graduação em Bioquímica, Instituto de Química, Universidade Federal do Rio de Janeiro (UFRJ), Rio de Janeiro 21941-909, Brazil; 3 Instituto Oswaldo Cruz (IOC), Fundação Oswaldo Cruz (FIOCRUZ), Rio de Janeiro 21040-900, Brazil; 4 Unidade Docente-Assistencial de Dermatologia, Universidade do Estado do Rio de Janeiro (UERJ), Rio de Janeiro 20551-030, Brazil; 5 Laboratório de Micologia, Hospital Universitário Pedro Ernesto (HUPE), Universidade do Estado do Rio de Janeiro (UERJ), Rio de Janeiro 20551-030, Brazil; 6 Rede Micologia RJ—Fundação de Amparo à Pesquisa do Estado do Rio de Janeiro (FAPERJ), Rio de Janeiro 21941-902, Brazil

**Keywords:** COVID-19, fungal infection, hydrolytic enzymes, biofilm formation, *Tenebrio molitor*

## Abstract

The pandemic caused by the severe acute respiratory syndrome coronavirus 2 (SARS-CoV-2) has been responsible for approximately 6.8 million deaths worldwide, threatening more than 753 million individuals. People with severe coronavirus disease-2019 (COVID-19) infection often exhibit an immunosuppression condition, resulting in greater chances of developing co-infections with bacteria and fungi, including opportunistic yeasts belonging to the *Saccharomyces* and *Candida* genera. In the present work, we have reported the case of a 75-year-old woman admitted at a Brazilian university hospital with an arterial ulcer in the left foot, which was being prepared for surgical amputation. The patient presented other underlying diseases and presented positive tests for COVID-19 prior to hospitalization. She received antimicrobial treatment, but her general condition worsened quickly, leading to death by septic shock after 4 days of hospitalization. Blood samples collected on the day she died were positive for yeast-like organisms, which were later identified as *Saccharomyces cerevisiae* by both biochemical and molecular methods. The fungal strain exhibited low minimal inhibitory concentration values for the antifungal agents tested (amphotericin B, 5-flucytosine, caspofungin, fluconazole and voriconazole), and it was able to produce important virulence factors, such as extracellular bioactive molecules (e.g., aspartic peptidase, phospholipase, esterase, phytase, catalase, hemolysin and siderophore) and biofilm. Despite the activity against planktonic cells, the antifungals were not able to impact the mature biofilm parameters (biomass and viability). Additionally, the *S. cerevisiae* strain caused the death of *Tenebrio molitor* larvae, depending on the fungal inoculum, and larvae immunosuppression with corticosteroids increased the larvae mortality rate. In conclusion, the present study highlighted the emergence of *S. cerevisiae* as an opportunistic fungal pathogen in immunosuppressed patients presenting several severe comorbidities, including COVID-19 infection.

## 1. Introduction

Starting 3 years ago, the coronavirus disease-2019 (COVID-19), caused by the severe acute respiratory syndrome coronavirus 2 (SARS-CoV-2), became a serious pandemic, which has resulted in more than 753 million confirmed cases worldwide, including over 6.8 million deaths (26 January 2023, https://covid19.who.int/). Patients with severe COVID-19 infection requiring hospitalization and intensive care may also be challenged to battle against coexisting infectious agents, which greatly worsens the patient’s clinical condition [1,2]. In fact, COVID-19 infection has been shown to lead patients to an immunosuppression status due to the immune dysregulation, which provides favorable conditions for opportunistic fungal infections. In this context, co-infections between SARS-CoV-2 and fungi, including yeasts (*Candida albicans*, non-*albicans Candida* species, *Cryptococcus neoformans* and *Saccharomyces cerevisiae*), dimorphic fungi (*Histoplasma capsulatum* and *Paracoccidioides* spp.) and molds (*Aspergillus* spp., *Mucor* spp. and *Rhizopus* spp.) have been reported [1,2].

The incidence of systemic infections caused by *S. cerevisiae*, which has been traditionally used in the agroalimentary industry for the production of wines, beers and foods by fermentation processes [3], has increased in recent years, particularly among immunosuppressed patients [3]. Infections by *S. cerevisiae* have been recently linked to the use of probiotics or dietary supplements, especially for critically ill patients in the intensive care unit (ICU) [4], including two reports in COVID-19 patients who received *Saccharomyces* supplementation prophylactically in the ICU after the development of diarrhea due to the use of several antimicrobials to treat bacterial co-infection with SARS-CoV-2 [5,6]. In this sense, *S. cerevisiae* should be considered an emerging opportunistic pathogen of low virulence [3].

In the present work, a clinical strain of *S. cerevisiae* recovered from the blood of a COVID-19-positive patient was used as an experimental model to evaluate the (i) susceptibility of both planktonic- and biofilm-forming cells to classical antifungal drugs, (ii) production of extracellular molecules (hydrolytic enzymes, siderophores and hemolysins) associated with fungal virulence, (iii) capacity to grow in different nutrient sources and (iv) ability to infect *Tenebrio molitor* larvae in vivo. 

## 2. Materials and Methods

### 2.1. Case Report

**Figure 1:**
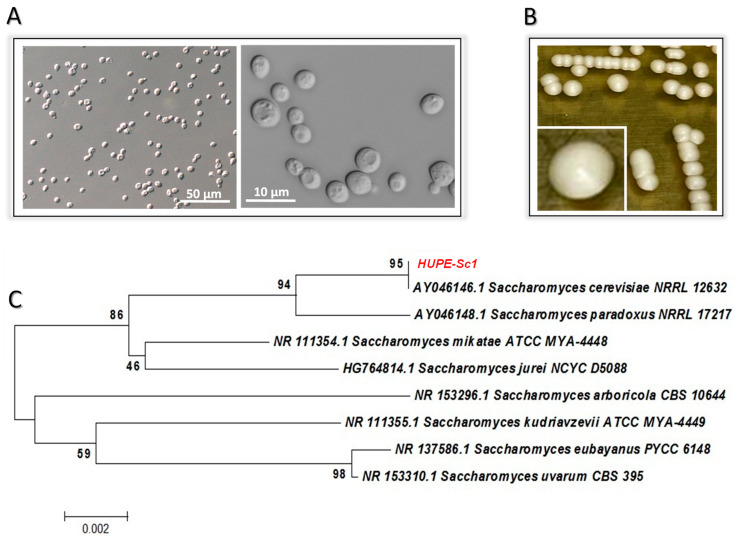
Microscopic (**A**) and macroscopic (**B**) aspects of *S. cerevisiae* HUPE-Sc1 strain grown for 48 h at 37 °C on Sabouraud dextrose agar. (**C**) Phylogenetic neighbor-joining dendrogram generated from a genetic similarity matrix based on comparison of *ITS1-5.8S-ITS2* gene sequences from HUPE-Sc1 strain and type strains belonging to the *Saccharomyces* genus; sequences were obtained from GenBank database.

A 75-year-old woman was admitted on 13 May 2021 to a University Hospital in Rio de Janeiro, Brazil, due to an arterial ulcer in the left foot (Fontaine IV Peripheral Arterial Disease). The disease started a year before and clinical treatments were unsuccessful, leading to bone exposure. Therefore, she was being prepared for a surgical amputation. Positive polymerase chain reaction results from two nasal swabs for SARS-CoV-2 was found twice, 30 and 10 days before admission, and she was treated symptomatically at home. Remarkable data in her past pathological history are systemic arterial hypertension, stroke with resulting aphasia and past history of smoking. She was started on intravenous antimicrobials (piperacillin and tazobactam) and heart monitoring due to the atrial fibrillation. Her general condition worsened quickly, showing drowsiness with disorientation, bradycardia and hypotension, metabolic acidosis, leukocytosis and elevated C-reactive protein. At that time, a nasal swab antigen test did not detect SARS-CoV-2. Two blood samples were collected and sent to the laboratory for culture approximately 6 hours before she died due to the septic shock, 4 days after hospitalization, on 17 May 2021. Yeast-like organisms were isolated from both blood culture samples (Figure 1A).

### 2.2. Yeast-like Identification 

The clinical yeast strain, designated as HUPE-Sc1, was cultured on Sabouraud dextrose agar (SDA; Difco, Becton, Dickinson and Company, La Jolla, CA, USA) plate at 37°C. Yeast identification was carried out by both phenotypic and molecular assays. Carbohydrate assimilation and metabolic enzymatic profiles were evaluated by VITEK 2^®^ system (bioMérieux, Marcy-l’Étoile, France) using the yeast (YST) card, according to the manufacturer’s guidelines. In parallel, amplification and sequencing of the *ITS1-5.8S-ITS2* gene were performed, and the amplicons were purified and sequences from both DNA strands were generated and edited with the Sequencher^TM^ version 4.9 (Gene Codes Corporation, Ann Arbor, MI, USA), followed by alignment using Mega version 4.0.2 software (https://www.megasoftware.net). Sequences corresponding to the *ITS* genes from S*accharomyces* spp. were obtained from the GenBank database (www.ncbi.nlm.nih.gov/genbank/).

### 2.3. Antifungal Susceptibility Assay

Antifungal susceptibility testing was performed according to the standardized broth microdilution technique described by Clinical & Laboratory Standards Institute (CLSI) in the document M27-A3 [7]. Antifungal drugs tested were amphotericin B, caspofungin, 5-flucytosine, fluconazole and voriconazole (Sigma-Aldrich, St Louis, MO, USA). The minimum inhibitory concentration (MIC) values of the drugs on planktonic yeast cells were determined according to the CLSI M27S3 protocol [8]. *Candida parapsilosis* ATCC 22019 and *Candida krusei* ATCC 6258 were used as quality control strains.

### 2.4. Detection of Extracellular Molecules

The production of extracellular molecules by fungal cells was carried out in agar plate assays. Briefly, the aspartic peptidase activity was determined using 1.17% yeast carbon base (YCB; Sigma-Aldrich, St Louis, MO, USA) medium supplemented with 1% bovine serum albumin (BSA; Sigma-Aldrich, St Louis, MO, USA) [9]. Caseinolytic activity was assessed using SDA containing 0.4% casein (Sigma-Aldrich, St Louis, MO, USA) [10]. Phospholipase activity was performed using egg yolk agar plate [11]. Esterase production was assayed using the Tween agar plate [12]. Phytase activity was evaluated using the calcium phytate agar [13]. Hemolysin production was evaluated by adding 7 mL of fresh sheep blood to 100 mL of SDA supplemented with 3% glucose [14]. Siderophore production was determined using blue indicator dye, chrome azurol S (CAS; Sigma-Aldrich, St Louis, MO, USA) [15,16]. To determine the production of these extracellular molecules, aliquots (10 µL) of 48 h old cultured fungal cells (10^7^ yeasts/mL) were spotted on the surface of each agar medium and incubated at 37 °C for up to 7 days. The colony diameter (a) and the diameter of the colony plus the hydrolysis/precipitation zone (b) were measured by a digital paquimeter and the production of each molecule was expressed as Pz value (a/b), as previously described [11]. *Candida haemulonii* (clinical isolate LIP*Ch*16) was used as positive control for detecting all the extracellular molecules investigated under the experimental conditions employed herein [16]. Additionally, catalase activity was also evaluated by the addition of 3% hydrogen peroxide (H_2_O_2_; Sigma-Aldrich, St Louis, MO, USA) to 10 μL of a fungal cell suspension in PBS containing 10^8^ yeasts/mL on glass slides. Catalase is an enzyme able to cause the hydrolysis of H_2_O_2_ in water and oxygen, and the release of oxygen results in the formation of bubbles that can be easily visualized.

### 2.5. Culture Supernatant Harvesting

*Saccharomyces cerevisiae* HUPE-Sc1 strain was grown in Sabouraud dextrose broth (SDB) (5 mL containing 10^6^ cells/mL) for 48 h at 37 °C and then this culture was transferred to 50 mL of the same medium and incubated for additional 48 h to achieve substantial growth. Afterwards, the culture was harvested by centrifugation (4000× *g*, 5 min, 4 °C), and the supernatant was filtered through a 0.22 µm membrane (Millipore, São Paulo, SP, Brazil). The cell-free supernatant was concentrated approximately 10 times in a 10,000 molecular weight cutoff AMICON micropartition system (AMICON, Beverly, MA, USA), and then the protein concentration was determined by the method described by Lowry and colleagues [17], using BSA as standard. During yeast growth, the cells release enzymes that support fungal nutrition and growth, such as peptidases, into the extracellular environment. In this sense, we evaluated the ability of the enzymes present in the culture supernatant of *S. cerevisiae* to cause hemolysis of fresh erythrocytes and the hydrolysis of hemoglobin, as described in the sections below.

### 2.6. Hemolysis Assay

The hemolytic activity from both yeast cells and cell-free culture supernatant of *S. cerevisiae* was assayed by incubation with sheep erythrocytes. The whole sheep blood (10 mL; Cultilab, Rio de Janeiro, RJ, Brazil) was centrifuged at 500× *g* for 10 min at 4 °C to isolate erythrocytes, followed by three washes with phosphate-buffered saline (PBS; pH 7.2) until the supernatant was clear. Then, the erythrocytes were resuspended in 10 mL of PBS. Afterwards, 100 μL of PBS containing different concentrations of yeasts (10^6^, 10^7^ and 10^8^ cells) and different protein amounts from fungal culture supernatant (2.5, 5 and 10 μg) were added to 100 μL of erythrocyte suspension (4%) in a 96-well plate at 37 °C for 3 and 24 h. After incubation, the supernatant was collected by centrifugation, and 100 μL was transferred to a new 96-well microtiter plate [18]. The absorbance at 415 nm was measured. A 0.1% Triton X-100 solution was used as positive control (100% lysis), and PBS was used as negative control [18].

### 2.7. Hemoglobin Hydrolysis

To investigate the ability of *S. cerevisiae*-secreted enzymes to hydrolyze hemoglobin, different protein amounts (2.5, 5 and 10 μg) of the culture supernatant were incubated with hemoglobin (20 μg) in PBS at 37°C for 1 or 24 h. The control systems were prepared in the same way, containing (i) hemoglobin (without the addition of culture supernatant) and (ii) each different amount of protein in the culture supernatant (without hemoglobin). After incubation, the samples were treated with an equal volume of sodium dodecyl sulfate-polyacrylamide gel electrophoresis (SDS-PAGE) sample buffer (125 mM Tris, pH 6.8, 4% SDS, 20% glycerol and 0.002% bromophenol blue) containing 10% β-mercaptoethanol, followed by heating at 100 °C for 5 min. Proteins were analyzed on 20% SDS-PAGE by the method described by Laemmli [19]. Electrophoresis was carried out at 120 V and 120 mA for 90 min at room temperature, and the gels were silver stained [20]. A clinical isolate of *C. haemulonii* (LIP*Ch*2) was used as positive control for this experiment [21].

### 2.8. Fungal Growth in Different Nutrient Sources

To evaluate the growth capability of *S. cerevisiae* in different nutrient sources, yeasts were grown overnight in SDB at 37 °C, washed twice with sterile PBS and suspended in the same buffer. Then, 10^4^ yeasts/mL were incubated for 24 h at 37 °C in four different conditions: SDB, sheep erythrocytes (100%), diluted sheep erythrocytes (2% in PBS) and fetal bovine serum (FBS). After incubation, the yeasts grown on each medium were washed in PBS, diluted and then 10 μL of the cell suspensions were plated onto SDA in order to determine the colony forming units (CFUs). Plates were incubated for 24 h at 37 °C and then the CFUs were counted. In parallel, aliquots (10 μL) of each system were spotted onto SDA before the incubation at 37 °C (time 0 h) and after 24 h of incubation. In both cases, plates were incubated at 37 °C for 24 h to allow fungal growth.

### 2.9. Biofilm Formation

To evaluate the biofilm formation, fungal suspensions in SDB (200 μL containing 10^6^ yeasts) were transferred into wells of 96-well polystyrene microtiter plates (Jet Biofil, Guangzhou, China) and then incubated without agitation at 37 °C up to 96 h. Medium-only blanks were also set up in parallel. After each time point, the supernatant fluids were carefully removed, and the wells were washed three times with PBS to remove nonadherent cells. Biofilm biomass quantification was assessed in a microplate reader (SpectraMax 190, Molecular Devices, Sunnyvale, CA, USA) at 590 nm after crystal violet incorporation (Sigma-Aldrich, St Louis, MO, USA) in methanol-fixed biofilm [22]. The metabolic activity of the biofilm was determined using a colorimetric assay, which measures at 492 nm the reduction of 2,3-bis (2-methoxy-4-nitro-5-sulfophenyl)-5-[(phenylamino) carbonyl]-2H-tetrazolium hydroxide (XTT; Sigma-Aldrich, St Louis, MO, USA) to a water-soluble brown formazan product [22]. The extracellular matrix was quantified at 530 nm after safranin impregnation in non-fixed biofilms [23]. The clinical isolate LIP*Ch*4 of *C. haemulonii* was used as positive control for this experiment [24].

### 2.10. Antifungal Susceptibility of Biofilm-Forming Cells

In this assay, the *S. cerevisiae* cells were incubated at 37 °C for 48 h to allow biofilm formation, as described above. Then, the supernatant was carefully removed, and the mature biofilm was washed once with sterile PBS. An aliquot of 200 μL of RPMI-1640 buffered with MOPS and supplemented with the antifungals prepared according to the CLSI M27-A3 [7] protocol was added. The plates were incubated at 37 °C for additional 48 h. Finally, crystal violet staining and XTT reduction assay were performed in order to evaluate biofilm biomass and viability parameters, respectively [22].

### 2.11. In Vivo Infection in Tenebrio molitor Larvae

*Tenebrio molitor* larvae exhibiting clear and uniform color and weighing between 70 and 100 mg were selected for the survival studies. For these experiments, *S. cerevisiae* yeasts were grown overnight in SDB at 37 °C, washed twice with sterile PBS and suspended in the same buffer. The curves were performed through injection of different fungal inocula (10^4^, 10^5^, 10^6^ and 10^7^ fungi/larva) to determine the appropriate concentration to be injected in the subsequent experiments. Larvae (10 per each assayed group) were inoculated with fungi using an insulin syringe (10 μL/larva) and incubated at 37 °C in Petri dishes containing rearing diet. The inoculation was performed by the injection of cell suspensions into the larvae hemocoel in the ventral portion at the second visible sternite above the legs [25]. Larvae inoculated with sterile PBS were used as control groups. Larvae were assessed daily, up to 7 days, to check their survival, being scored as dead when they displayed no movement in response to touch. Experiments were performed in triplicate with 10 larvae per group, totaling 30 animals per group, which were used to construct the survival curve [25].

### 2.12. Impact of Immunosuppression on Larvae Survival

To evaluate the effect of corticosteroid immunosuppression on *T. molitor* survival, each larva was exposed to 100 μg of methylprednisolone acetate (40 mg/mL stock solution in water) and infected with 10^6^ yeasts of *S. cerevisiae*. Control groups were composed by larvae inoculated only with (i) PBS, (ii) 100 μg of methylprednisolone acetate and (iii) 10^6^ yeasts of *S. cerevisiae*. Larvae rearing and incubation conditions used were the same described in the section above [26].

### 2.13. Statistics

Experiments were performed in triplicate, in three independent experimental sets, and data were expressed as mean ± standard deviation. The results were evaluated by analysis of variance (one-way ANOVA) and Tukey’s multiple comparison test or Dunnett’s multiple comparison test using GraphPad Prism 8 computer software. Survival analyses were determined using the long-rank test and the Kaplan–Meier curves on GraphPad Prism 8 software. In all analyses, *p* values of 0.05 or less were considered statistically significant.

## 3. Results

### 3.1. Yeast Identification by Biochemical and Molecular Approaches

The yeast-like fungal isolate strain HUPE-Sc1 (Figure 1A) was identified by mycology methodologies. Firstly, the fungal isolate developed a white color after 48 h of incubation on SDA (Figure 1B). The carbohydrate assimilation and metabolic enzymatic profiles evaluated with VITEK 2^®^ automated system identified it as *S. cerevisiae* with a probability of identity of 96% and, in this regard, three contradictory tests were detected: d-maltose assimilation (dMALa), d-trehalose assimilation (dTREa) and d-lactate assimilation (LATa). In parallel, PCR followed by sequencing of the *ITS* gene was used as the gold standard for the precise identification of this fungal isolate. The *ITS* sequencing alignment scores of the HUPE-Sc1 strain exhibited 100% identity compared with corresponding *ITS* sequence from a reference *S. cerevisiae* strain deposited in GenBank (Figure 1C). The *ITS* sequences obtained during this study were deposited in GenBank under the accession number OQ030183.

### 3.2. Susceptibility Profile to Antifungal Agents

As no standardized interpretative criterion to determine susceptibility has been established until now for the *Saccharomyces* species, breakpoints adopted for *Candida* species have previously guided interpretation. In the present study, the assayed antifungals (amphotericin B, 5-flucytosine, caspofungin, fluconazole and voriconazole) showed greater activity against the *S. cerevisiae* HUPE-Sc1 strain with low MIC values (Table 1).

### 3.3. Production of Biologically Active Extracellular Molecules and Growth in Different Nutrient Sources

**Figure 2:**
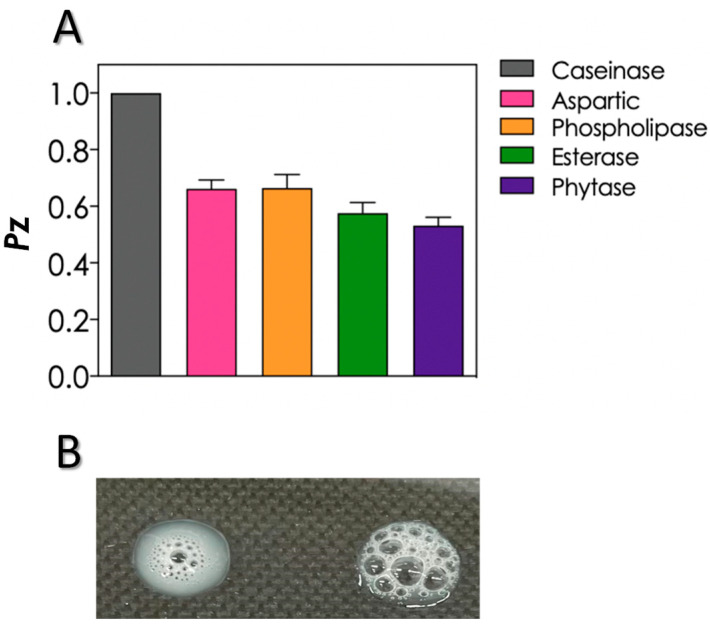
Extracellular molecules produced by *S. cerevisiae* HUPE-Sc1 strain by means of agar plates containing specific substrates to detect: caseinase, aspartic peptidase, phospholipase, esterase and phytase (**A**), and catalase activity demonstrated by the formation of bubbles after the contact of *S. cerevisiae* HUPE-Sc1 strain with H_2_O_2_, which correspond to the hydrolysis of H_2_O_2_ in water and molecular oxygen (**B**). The results represent means ± standard deviation of three independent experiments.

The production of biologically active extracellular molecules associated with fungal virulence was evaluated using the classical plate method containing specific substrates. In this sense, the *S. cerevisiae* HUPE-Sc1 strain was able to secrete different classes of hydrolytic enzymes, including aspartic peptidase, phospholipase, esterase and phytase (Figure 2A). The Pz values classified HUPE-Sc1 strain as a good producer of these extracellular molecules, as proposed by Price et al. [11]. In contrast, caseinolytic activity was not detected under the employed experimental conditions (Figure 2A). Additionally, the HUPE-Sc1 strain also produced catalase activity as can be seen by the formation of bubbles, which correspond to the hydrolysis of H_2_O_2_ in water and molecular oxygen when the fungal cells were exposed to H_2_O_2_ (Figure 2B).

**Figure 3:**
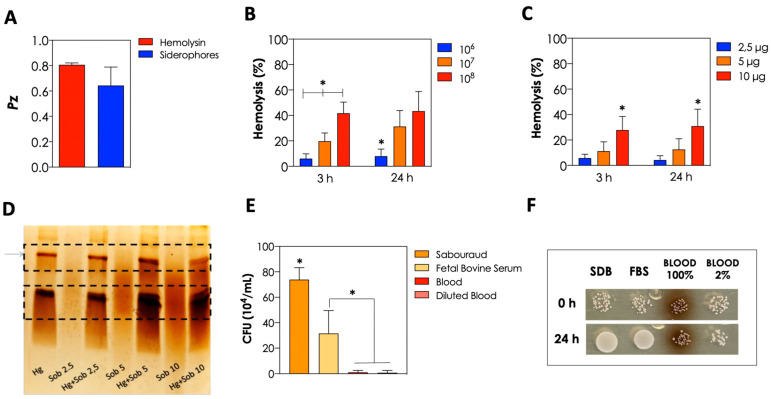
Extracellular molecules produced by *S. cerevisiae* HUPE-Sc1 strain by means of agar plates containing specific substrates to detect hemolysin and siderophores (**A**). Lysis of fresh erythrocytes after 3 h and 24 h of incubation at 37 °C by different inocula of *S. cerevisiae* HUPE-Sc1 strain (10^6^, 10^7^ and 10^8^ cells) (**B**) and different protein amounts of the cell-free culture supernatant in Sabouraud (**C**). SDS-PAGE demonstrating the hydrolysis of hemoglobin (Hg) by the enzymes present in the culture supernatant (Sob) of HUPE-Sc1 strain (2.5, 5 and 10 μg of proteins); the dashed boxes highlight intact hemoglobin (upper box) and hydrolyzed hemoglobin (lower box) (**D**). HUPE-Sc1 strain growth capacity in different nutrient sources (Sabouraud, FBS, blood [100%] and diluted blood [2%]) after incubation of 10^4^ yeasts/mL at 37 °C for 24 h as demonstrated by CFU counts (**E**) and by spot inoculum of 10 μL of each system on SDA (**F**). The results represent means ± standard deviation of three independent experiments. The symbol (*) indicates *p* values < 0.05 (one-way ANOVA, Tukey’s multiple comparison).

The *S. cerevisiae* HUPE-Sc1 strain was also able to produce other two important extracellular molecules: hemolysin and siderophores (Figure 3A), exhibiting weak and good activities, respectively, according to the Pz classification. Once the strain was obtained from blood and demonstrated hemolytic activity, we evaluated its ability to lyse fresh erythrocytes. In this sense, the in-solution co-incubation of fresh erythrocytes and *S. cerevisiae* yeast cells induced the hemolysis in a typical fungal-concentration-dependent way, but no differences were observed regarding the incubation time, being the hemolytic activity after 3 h and 24 h of incubation almost the same (Figure 3B). Additionally, we evaluated the ability of the enzymes present in the cell-free culture supernatant of the HUPE-Sc1 strain to lyse fresh erythrocytes and, similar to planktonic cells, we also observed that the hemolysis occurred in a dose-dependent but not time-dependent manner (Figure 3C). Additionally, the enzymes (belonging to the peptidase class) present in the culture supernatant of HUPE-Sc1 strain were also able to hydrolyze hemoglobin in a dose-dependent manner, as demonstrated by SDS-PAGE (Figure 3D). Nonetheless, the growth ability of the HUPE-Sc1 strain in FBS and blood (non-diluted and diluted) was considerably reduced in comparison with SDB, but the yeast cells were able to survive in all tested sources (Figure 3E,F).

### 3.4. Biofilm Formation and Impact of Antifungals on Mature Biofilms

**Figure 4:**
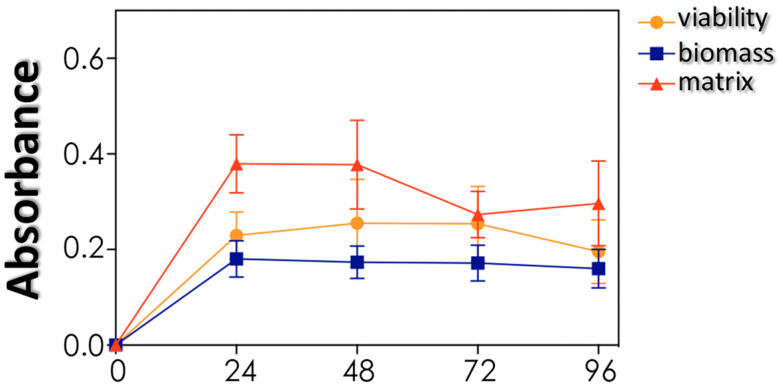
Biofilm formation by *S. cerevisiae* HUPE-Sc1 strain over a polystyrene surface. Three biofilm parameters were measured: biomass (absorbance at 590 nm), viability (absorbance at 492 nm) and extracellular matrix (absorbance at 530 nm). The results represent means ± standard deviation of three independent experiments.

The capacity to form biofilm was also evaluated, since biofilm is considered a multifunctional structure with both virulence and resistance properties. *S. cerevisiae* HUPE-Sc1 strain adhered to polystyrene surface forming a classical and viable biofilm structure, as observed by means of quantification of three parameters: biomass, viability and extracellular matrix production (Figure 4).

**Figure 5:**
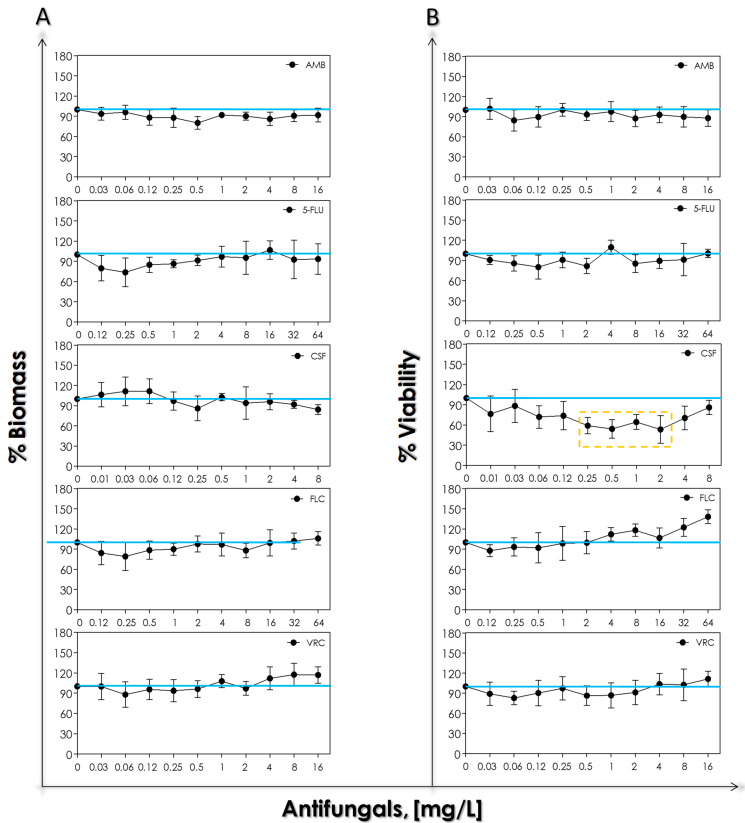
Biomass (**A**) and viability (**B**) of biofilm formed by *S. cerevisiae* HUPE-Sc1 strain over a polystyrene surface exposed to different concentrations of antifungals: amphotericin B (AMB), 5-flucytosine (5-FLU), caspofungin (CSF), fluconazole (FLC) and voriconazole (VRC). The results were assessed spectroscopically (biomass at 590 nm and viability at 492 nm) and expressed as the mean of metabolic and biomass percentages compared to untreated biofilms (control), which correspond to 100% (blue line). The graphs exhibit the mean ± standard deviation of three independent experiments. The dashed box represents the concentrations of CSF that caused statistically significant reduction in cell viability in relation to the respective control (*p* < 0.05; one-way ANOVA analysis of variance and Dunnett’s multiple comparison test).

In order to evaluate the antifungal susceptibility profile of mature biofilm-forming *S. cerevisiae* cells, mature biofilms were incubated for 48 h with different concentrations of antifungals. Our results revealed that none of the tested antifungals were able to significantly reduce the biofilm biomass (Figure 5A). Regarding the biofilm viability, only caspofungin reduced the cellular metabolic activity at concentrations ranging from 0.25 to 2 mg/L (approximately 40%), while at higher caspofungin concentrations, a classical paradoxical effect was observed (Figure 5B).

### 3.5. Mortality of T. molitor Larvae Infected with S. cerevisiae and Impact of Immunosuppression on Larvae

**Figure 6:**
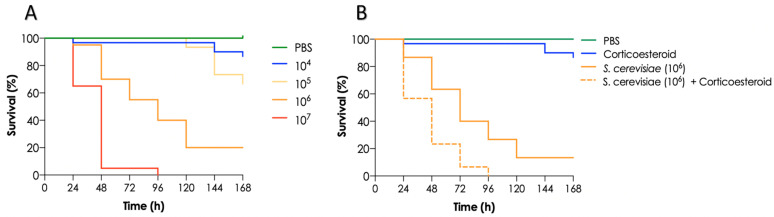
Survival curves of *T. molitor* larvae infected with different inoculum sizes of *S. cerevisiae* HUPE-Sc1 strain (10^4^, 10^5^, 10^6^ and 10^7^ fungi/larvae) (**A**) and effect of the treatment with corticosteroids (100 μg) on larvae survival after injection of 10^6^ fungi/larva (**B**). In both cases, groups of 10 larvae were infected with indicated systems, repeated three times and pooled together in order to build survival curves with 30 animals. Negative controls were composed by *T. molitor* larvae injected only with PBS or corticosteroids (100 μg).

The in vivo virulence of the *S. cerevisiae* HUPE-Sc1 strain was investigated using the invertebrate model of *T. molitor* larvae. In this context, larvae mortality rate increased as the fungal inoculum increased as well: 90% of larvae infected with 10^4^ yeasts survived after 7 days post-infection, 70% of larvae infected with 10^5^ yeasts survived after the same incubation time, and only 20% of larvae infected with 10^6^ yeasts survived, while all larvae infected with 10^7^ yeasts died after 96 h post-infection (Figure 6A). As *S. cerevisiae* is considered an opportunistic fungus generally causing infection in immunocompromised patients, we treated the larvae with corticosteroids, resulting in an immunosuppression condition. In this sense, we observed that the corticosteroid treatment significantly increased (*p* < 0.0001; long rank-rank [Mantel–Cox] test) the larvae mortality rate at the fungal cell density used (10^6^ cells/larvae) in comparison with non-treated larvae (Figure 6B). Regarding the negative controls, all larvae survived the injection with PBS and 90% survived the treatment with corticosteroids. 

## 4. Discussion

It is well known that viral infections, such as mononucleosis, chikungunya and dengue, for example, can lead to several months of immunosuppression in a previously healthy patient. Therefore, it is possible that the previous COVID-19 led to an immunosuppression in a patient with peripheral arterial disease, exposing her to an opportunistic fungal disease. The fast progression to septic shock with the isolation of *S. cerevisiae* in the bloodstream favors the link of a recent COVID-19 infection followed by fungal sepsis. The *S. cerevisiae* HUPE-Sc1 strain showed low MICs for the commonly used antifungals for serious systemic invasive infections. Unfortunately, the diagnosis of sepsis was made too late due to the rapid general health deterioration of the patient. Opportunistic invasive fungal infections should be kept in the mind of all emergency or intensive care unit doctors.

Indeed, a recent report of bloodstream infection caused by *S. cerevisiae* in two elderly patients with COVID -19 hospitalized in an ICU in Greece after receiving supplementation due to the diarrhea highlighted the importance of caution while using probiotic preparations in COVID-19 patients [5]. Although the patients had other complications caused by the COVID-19 infection, the treatment first with anidulafungin and then with fluconazole resulted in the resolution of the fungal infection [5]. The in vitro susceptibility tests suggested that the isolates were susceptible to fluconazole, voriconazole, posaconazole, amphotericin B, anifulafungin and 5-flucytosine [5]. Additionally, in Brazil, another COVID-19 patient also developed fungemia by *S. cerevisiae* after supplementation with *Saccharomyces* due to the diarrhea, which could be facilitated by the antibiotic regimen he was going through in addition to the use of vasoactive amines and the intestinal damage commonly caused by SARS-CoV-2 [6]. Fluconazole treatment was sufficient for the resolution of fungal infection, but the patient died due to the pulmonary infection and other complications caused by the COVID-19 infection [6].

As mentioned above, the *S. cerevisiae* HUPE-Sc1 strain exhibited low MICs to amphotericin B, caspofungin, 5-flucytosine, fluconazole and voriconazole, which is in accordance with literature reports that demonstrate that *S. cerevisiae* is usually susceptible to the main antifungal classes used in clinical practice, with the exception of fluconazole, for which variable susceptibility profiles have been described over the years [27,28,29,30]. Similar to our results, Pérez-Cantero and coworkers [27] and Echeverría-Irigoyen and coworkers [29] reported excellent in vitro antifungal activity of 5-flucytosine against *S. cerevisiae*. Indeed, the current guidelines recommend the use of amphotericin B or amphotericin B in combination with 5-flucytosine to treat severe cases of infections caused by *S. cerevisiae* [27]. Unfortunately, 5-fluocitosine is not commercially available in Brazil. Echinocandins are also recommended as treatment options for *S. cerevisiae* infections, but despite in vitro susceptibility, caspofungin treatment in a pediatric surgical ICU patient with respiratory distress did not result in a good clinical response, the caspofungin treatment being switched to liposomal amphotericin B, resulting in the cure of *S. cerevisiae* infection [4]. As reviewed by the authors, amphotericin B or liposomal amphotericin B is frequently effective against *S. cerevisiae* fungemia in pediatric patients [4]. 

Extracellular enzymes are known as important virulence factors produced by different *Candida* species and have also been described in *S. cerevisiae* strains. Herein, we described that the *S. cerevisiae* HUPE-Sc1 strain was able to produce aspartic peptidase, phospholipase, esterase and phytase. Reports of *S. cerevisiae* production of aspartic peptidase and phospholipase can be found in the literature, both in clinical and industrial strains [31]. In this sense, Llanos and coworkers [31] observed that 81% and 96% of industrial and clinical isolates of *S. cerevisiae*, respectively, were moderate/high producers of aspartic peptidases, while the remaining isolates presented weak activity. The same authors also demonstrated that clinical isolates of *S. cerevisiae* were able to produce higher amounts of phospholipase than industrial isolates, with approximately 85% of clinical isolates being moderate/high producers, while almost 48% of industrial strains were low producers; the same percentage was considered moderate producers of phospholipase [31]. These results suggest that phospholipase activity can be related in some level to *S. cerevisiae* virulence. Corroborating these results, Irme and coworkers [32] also demonstrated that non-mycoses *S. cerevisiae* isolates produced lower phospholipase activity than clinical isolates of the same species. 

Phytase is an enzyme responsible for the hydrolysis of phytic acid into inorganic phosphate and inositol [13]. Indeed, phytate is the main reservoir of phosphorous in plants and, consequently, is common in animal and human diets. In this context, phytase seems to contribute to survival of microorganisms in the gastrointestinal tract where nutrients are scarce [13]. Herein, we demonstrated that our clinical isolate of *S. cerevisiae* was a good producer of phytase, which could play a role in the infectious process. Additionally, it is worth mentioning that phytate is considered an anti-nutrient factor in diets due to its ability to chelate minerals such as calcium, zinc and iron, and, additionally, it binds to proteins and lipids, resulting in the reduction of intestinal absorption of these nutrients, being particularly a problem for animal diets [33]. In this sense, it is common the use of inorganic phosphorus to supplement the diet of pigs, poultry and fish, but the phosphorus not used by the organism is excreted into the environment, resulting in environmental problems. Strains of *S. cerevisiae* have already been used in biotechnological processes to optimize phytase production, which can be used to reduce the phytate in animal feed and improve the bioavailability of phosphorus in monogastric animals [33]. We also observed that the *S. cerevisiae* HUPE-Sc1 strain produced catalase, which is an enzyme known to offer protection against oxidative damage caused by H_2_O_2_ [34]. Indeed, the yeast *S. cerevisiae* is used as a model of eukaryotic cell to study oxidative stress responses.

Hemolytic activity is considered an important virulence factor described in many *Candida* species and not often common in environmental strains of *S. cerevisiae* [35]. In our work, we demonstrated that our clinical isolate was able to produce hemolytic activity by two different methodologies: through supplementation of SDA with blood and through the co-incubation of the yeasts with fresh sheep blood, demonstrating the virulence potential of this strain to lyse erythrocytes and obtain nutrients when it reaches the bloodstream of vulnerable individuals. Imre and coworkers [32] demonstrated that commercial, non-mycosis and mycosis isolates of *S. cerevisiae* were able to produce both α- and β-hemolytic activity, but the mycoses’ isolates exhibited higher β-hemolytic activity when compared with the other strains. Additionally, we also demonstrated that enzymes released into the extracellular environment by the *S. cerevisiae* HUPE-Sc1 strain were able to hydrolyze hemoglobin, which corroborates its potential to acquire nutrients in the bloodstream of susceptible individuals.

Biofilm formation by *S. cerevisiae* is a well-known event, being characterized as a thin layer of yeast cells surrounded by an extracellular matrix with low density [36]. Indeed, *S. cerevisiae* has been used as a model of yeast to study the development of biofilm and its regulation using molecular tools [36]. Similar to us, Bojsen and coworkers [36] demonstrated that voriconazole, caspofungin and 5-flucytosine have no action on the mature biofilm of *S. cerevisiae*; on the other hand, they demonstrated that amphotericin B was the only antifungal used able to kill the biofilm-forming cells, but in our study amphotericin B did not impact neither the cell viability nor the biomass of *S. cerevisiae* biofilm. Those authors also demonstrated that antifungal susceptibility is dependent on the growth phase of both planktonic and biofilm cells, being the response against antifungals better in exponentially growing planktonic cells and in growing biofilms than in non-growing planktonic cells and in mature biofilms [36]. In this sense, the presence of biofilms is an aggravating factor for the successful treatment of hospitalized patients, representing a challenge to clinicians especially in the case of the opportunistic infections that generally affect individuals with other underlying diseases.

The use of in vivo models is undoubtedly necessary to the progress of scientific research, including the study of the basis of microbial pathogenicity and also the development of vaccines and new therapies for a wide range of diseases [37]. This historically included mainly vertebrate model of animals, which resulted in ethical concerns due to the distress, pain and sacrifice of animals used in experimental research, leading to the approval of laws to regulate animal use for research purposes [37]. Furthermore, animal use also requires more economic investment and qualified people, making the process more complex and expensive to maintain. To overcome these problems, the use of invertebrate models increased considerably in the last decade, avoiding ethical issues and high financial investments. In this scenario, insects were found to be a good host model for the study of microbial infections since they possess an innate immune system similar to vertebrates, a short lifecycle and allow the conduction of large-scale experiments [37].

In this regard, *T. molitor* larvae, known as the mealworm, have been used recently as a host model for microbial infections studies, including bacteria, yeasts and filamentous fungi. A recent study using the yeasts *C. albicans* and *C. neoformans* demonstrated that an increase of the fungal inoculum injected into the larvae resulted in increasing mortality rates [25]. Herein, we observed the same pattern when infecting *T. molitor* larvae with our clinical strain of *S. cerevisiae*. Additionally, we demonstrated that larvae treated with corticosteroid and infected with *S. cerevisiae* exhibited a higher mortality rate when compared with larvae only infected by the yeasts, indicating a possible immunosuppression of larvae. However, to our knowledge, no previous studies regarding *T. molitor* larvae immunosuppression was conducted until now and more experiments are necessary to clarify these issues. 

## 5. Conclusions

In conclusion, the present study highlighted the emergence of *S. cerevisiae* as an opportunistic pathogen with no resistance profile to antifungal agents commonly used in clinical practice, but with ability to produce important virulence factors that could facilitate the establishment of the infectious process in vulnerable individuals, contributing to the worsening of the health status of patients that are often experiencing other infections, as happened with the patient with COVID-19 infection reported in the present work.

## Figures and Tables

**Table 1:** Antifungal susceptibility profile of *S. cerevisiae* clinical isolate studied herein.

Antifungals	MIC *, [mg/L]
Amphotericin B	0.25
5-Flucytosine	<0.125
Caspofungin	0.5
Fluconazole	1
Voriconazole	<0.03

* Minimal inhibitory concentration.

## Data Availability

Not applicable.
